# Low temperature-fired Ni-Cu-Zn ferrite nanoparticles through auto-combustion method for multilayer chip inductor applications

**DOI:** 10.1186/1556-276X-7-112

**Published:** 2012-02-08

**Authors:** Khalid Mujasam Batoo, Mohammad Shahnawaze Ansari

**Affiliations:** 1 King Abdullah Institute for Nanotechnology, King Saud University, PO Box 2460, Riyadh, 11451, Kingdom of Saudi Arabia; 2 Centre of Nanotechnology, King Abdulaziz University, PO Box 80216, Jeddah, 21589, Kingdom of Saudi Arabia

**Keywords:** nanoparticles, ferrites, dielectric constant, ac conductivity, impedance spectroscopy.

## Abstract

Ferrite nanoparticles of basic composition Ni_0.7-*x*_Zn_*x*_Cu_0.3_Fe_2_O_4 _(0.0 ≤ *x *≤ 0.2, *x *= 0.05) were synthesized through auto-combustion method and were characterized for structural properties using X-ray diffraction [XRD], scanning electron microscopy, transmission electron microscopy, and Fourier transform infrared spectroscopy [FT-IR]. XRD analysis of the powder samples sintered at 600°C for 4 h showed the cubic spinel structure for ferrites with a narrow size distribution from 28 to 32 nm. FT-IR showed two absorption bands (*v*_1 _and *v*_2_) that are attributed to the stretching vibration of tetrahedral and octahedral sites. The effect of Zn doping on the electrical properties was studied using dielectric and impedance spectroscopy at room temperature. The dielectric parameters (*ε*', *ε*″, tan*δ*, and *σ*_ac_) show their maximum value for 10% Zn doping. The dielectric constant and loss tangent decrease with increasing frequency of the applied field. The results are explained in the light of dielectric polarization which is similar to the conduction phenomenon. The complex impedance shows that the conduction process in grown nanoparticles takes place predominantly through grain boundary volume.

**PACS**: 75.50.Gg; 78.20; 77.22.Gm.

## Introduction

The study of ferrites has attracted immense attention of the scientific community because of their novel properties and technological applications especially when the size of the particles approaches to nanometer scale. More novel electrical and magnetic behaviors have been observed in comparison with their bulk counterpart [1,2]. In general, the transport properties of the nanomaterials are predominantly controlled by the grain boundaries than by the grain itself [3]. Due to this reason, the magnetic materials have explored a wide range of applications and thus are replacing conventional materials.

In the last two decades, latest advancement in wireless technology has explored the area of real-time communication. Internet-accessible cell phones and high-speed wireless local area network are the best examples of this technology. The core of these systems is based on a radio frequency [RF] circuit consisting of transmission and receiving circuit blocks required in signal amplification, filtering, and modulation that in turn require hundreds of passive chip components such as capacitors and inductors. Inductors adapted to RF circuits of mobile devices are mostly multilayer chip inductors [MLCIs] and microspiral inductors. MLCIs were developed in the 1980s by thick film printing and co-firing technologies using low temperature-sintered Ni-Cu-Zn ferrite and Ag. Recently, Ni-Cu-Zn ferrites have been developed to meet a demand for miniaturization of electronic components [4,5]. The ferrite powder needs to be sintered below 950°C in order to co-heat with silver internal electrodes (*T*_m _approximately 962°C) and should have low dielectric constants for MLCI application. Materials with high permeability are also required for reducing the number of layers in MLCIs and for realizing the better miniaturization [6]. Further, ferrite nanoparticles are commercially important for several applications such as in electromagnetic devices operating at radio frequencies where the superparamagnetic [SPM] properties have a strong influence in enhancing their quality of applications [7-9]. Nanoparticles of these materials exhibit interesting phase transitions from SPM state to ferri/ferromagnetic state or vice versa with a variation of temperature depending on their sizes. In this ferrite nanoparticle system, the Cu content of the compositions was kept constant at 30 at.% of the A site (AB_2_O_4 _spinel); nonmagnetic Zn^2+ ^ions occupy the tetrahedral A sites, replacing Fe^3+ ^ions, which eventually go to octahedral B sites. Hence, zinc cations magnetically dilute the system by making the A-B exchange interaction relatively weaker. This weaker coupling reduces the anisotropy energy of the system, which facilitates the onset of SPM relaxation in bigger size particles even at room temperature. Many reports are available in the literature on Ni-Cu ferrites where people have reported various properties of the studied ferrite material in bulk as well as in nanoscale form. Chakrabarti et al. [10] studied the magnetic properties of nanocrystalline Ni_0.2_Zn_0.6_Cu_0.2_Fe_2_O_4 _prepared using a chemical route method, and they reported that below 80 K, the nanoparticles exhibit superparamagnetism, and the saturation magnetization increases with increasing particle size. Seong et al. [11] investigated the structural and electrical properties of Cu-substituted Ni-Zn ferrites, and they have reported that the alternating current [ac] conductivity increases with increasing temperature of the sample and frequency of the applied field. Roy et al. [12] reported the effect of Mg substitution on electromagnetic properties of (Ni_0.25_Cu_0.20_Zn_0.55_)Fe_2_O_4 _ferrite prepared through auto-combustion method, and they found that the permeability and ac resistivity increased while the magnetic loss decreased with the progressive substitution. Jadhav et al. [13] reported the structural, electrical, and magnetic properties of Ni-Cu-Zn ferrite synthesized by citrate precursor method, and they reported that the dielectric properties (*ε*' and tan*δ*) decreases with increasing frequency of the applied field. They further report that the maximum value of the saturation magnetization was found for 20% Cu doping.

However, as per our best search, we have not found any detailed report in the literature on the dielectric and impedance properties of Zn-doped Ni_0.7-*x*_Cu_0.3_Fe_2_O_4 _ferrite nanoparticles. Therefore, keeping in view the high demand and importance of magnetic ferrite nanoparticles, we report in this paper the effect of Zn doping on the structural, cationic distribution, and conductivity properties of nanocrystalline Ni-Cu-Zn ferrites.

## Experimental details

### Material preparation

Nanoparticles of Ni_0.7-*x*_Zn_*x*_Cu_0.3_Fe_2_O_4 _(0.0 ≤ *x *≤ 0.2, *x *= 0.05) were prepared by auto-combustion method using 'AR' grade Ni(NO_3_)_2_.6H_2_O, CuCl, Zn(NO_3_)_2_.6H_2_O, and Fe(NO_3_)_2_.9H_2_O as raw materials. The stoichiometric mixtures of the mentioned materials were dissolved in deionized water, and few drops of ethyl alcohol were added to it. The solution was allowed for gel formation on the magnetic stirrer at 65°C with constant stirring. The gel formed was annealed at 200°C for 24 h, followed by grinding for 0.5 h. The dried gel was allowed to burn in a self-propagating combustion manner until the whole gel was completely burnt out to form a fluffy loose powder. The formed powder was heated for 4 h at 600°C to remove any organic material present while maintaining the rate of heating and cooling at 5°C/min and then finally ground for 0.5 h.

X-ray diffraction [XRD] (PANalytical X'Pert Pro, Almelo, The Netherlands) with CuK*α *(*λ *= 1.54 Å) was used to study the single-phase nature and nano-phase formation of the pure and doped Ni-Cu-Zn ferrite nanoparticles at room temperature. The microstructural analysis of the samples was carried out by field emission scanning electron microscopy [FESEM] (JSM 7600F, JEOL Ltd., Akishima, Tokyo, Japan) and field emission transmission electron microscopy [FETEM] (Jeol 2010, Tokyo, Japan). The infrared spectra of the powders (as pellets in KBr) were recorded by Fourier transform infrared spectrometry [FT-IR] (PerkinElmer Instruments, Waltham, MA, USA) in the range of 400 to 1,000 cm^-1 ^with a resolution of 1 cm^-1^.

The samples were pressed into circular disk-shaped pellets, and silver coating was done on the opposite faces to make parallel plate capacitor geometry with ferrite material as the dielectric medium. The dielectric and impedance spectroscopy measurements were performed in the frequency range of 42 Hz to 5 MHz using LCR HI-Tester (HIOKI 3532-50, HIOKI E.E. Corporation, Nagano, Japan).

## Results and discussion

### Structural characterization

**Figure 1:**
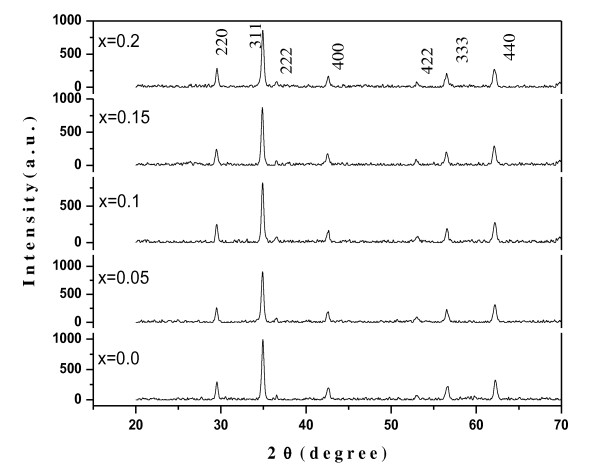
**XRD pattern of powdered Ni_0.7-*x*_Cu_0.3_Zn_*x*_Fe_2_O_4 _(0.0 ≤ *x *≤ 0.2, *x *= 0.05) ferrite nanoparticles**.

XRD patterns of the sintered Ni_0.7-*x*_Zn_*x*_Cu_0.3_Fe_2_O_4 _ferrites are shown in Figure 1. The most intense peaks in all specimens, indexed as (220), (311), (222), (400), (422), (333), and (440), are found to match well with single-phase cubic spinel (JCPS card number 08-0234). No additional phase corresponding to any structure in starting and doped samples was detected.

The lattice parameter of the samples was determined using this relation [14]:

(1) $$a_{\text{exp}}\text{ = }d_{hkl}\sqrt{h^{2}}+k^{2}+l^{2}.$$

The average crystallite size was determined from the diffraction peak broadening with the use of the Scherrer's equation [15]:

(2) $$t=\frac{0.98\lambda }{\beta \text{cos}\theta }.$$

Here, *λ *is the wavelength of the CuK*α *radiation (*λ *= 1.54060), and *β *is the full width at half maximum in radians.

The lattice parameters and crystallite sizes of the sintered ferrite specimens, evaluated by XRD analysis, are shown in Table 1 along with other structural parameters. It is seen that the grown ferrite samples show a narrow size distribution ranging from 28 to 32 nm. Also, the lattice constant is found to increase from 8.361 to 8.368 Å with increasing Zn content. The lattice parameter values are in expected range with the lattice parameters of spinel cubic ferrites [16,17]. The behavior can be attributed to the mismatching of ionic radii, where the ionic radius of Zn^2+ ^ion (0.84 Å) is larger than that of Ni^3+ ^ion (0.74 Å).

**Table 1:** Grain size, lattice parameters, and ionic radii data of Ni_0.7-*x*_Cu_0.3_Zn_*x*_Fe_2_O_4 _ferrite nanoparticles

Zn content	Grain size	Lattice constant		Ionic radii	
(*x*)	*D *(nm)	*a*_exp _(Å)	*a*_th _(Å)	*r*_A _(Å)	*r*_B _(Å)
0.00	28.2	8.361	8.359	0.680	0.755
0.05	28.6	8.363	8.362	0.682	0.755
0.10	29.8	8.365	8.363	0.683	0.755
0.15	30.5	8.367	8.365	0.685	0.755
0.20	31.3	8.368	8.366	0.688	0.755

The theoretical lattice parameter (*a*_th_) can then be calculated using this equation [14]:

(3) $$a_{\text{th}}\text{ = }\frac{8}{3\sqrt{3}}\left[\left(r_{\text{A}}+r_{\text{B}}\right)+\sqrt{3}\left(r_{\text{B}}+r_{\text{o}}\right)\right],$$

where *r*_o _is the radius of the oxygen ion (0.138 nm), and *r*_A _and *r*_B _are the ionic radii of tetrahedral (A) and octahedral (B) sites, respectively. The values of *r*_A _and *r*_B _will depend critically on the cation distribution of the given system. To calculate for *r*_A _and *r*_B_, the following cation distribution is proposed:

(4) $$\left({\text{Zn}}_{x}^{2+},{\text{ Fe}}_{1-\delta }^{3+}\right)\left[{\text{Ni}}_{0.7-x}^{2+},{\text{ Fe}}_{1+\delta }^{3+},{\text{ Fe}}_{\delta }^{2+}\right]{\text{O}}_{4}^{-}.$$

This cation distribution is based on the following:

1. NiFe_2_O_4 _and CuFe_2_O_4 _[18,19] are both inverse spinel in structure in which half of the ferric ions preferentially occupy the tetrahedral (A sites) and the other half occupy the octahedral sites (B sites).

2. On the other hand, Zn ions prefer to occupy the tetrahedral sites [20].

3. During the sintering process, oxygen loss occurs, leading a part of Fe^3+ ^ions to transform to Fe^2+ ^for charge compensation [14].

The data in Table 1 reveals that the values of the theoretical lattice parameter (*a*_th_), calculated assuming the suggested cation distribution formula, agree well with those experimentally obtained (*a*_exp_).

The mean radius of the ions at the tetrahedral site *r*_tetr _and the octahedral site *r*_oct _has been calculated according to these equations [21]:

(5) $$r_{\text{tetra}}\text{ = }a\sqrt{3}\left(\mu -0.25\right)-R_{\text{o}}$$

and

(6) $$r_{\text{oct}}\text{ = }a\left(\frac{5}{8}-\mu \right)-R_{\text{o}},$$

where *R*_o _is the radius of the oxygen ion (*R*_o _= 1.26 Å), and *μ *is the oxygen parameter. Here, we have taken the value *μ *= 0.375 by assuming that the spinel structure is not deformed by Zn^2+ ^doping [22,23].

The variation of X-ray density *D_hkl _*(theoretical), apparent density (experimental) *D_x_*, and porosity *P *as a function of Zn^2+ ^ion concentration (*x*) is reported in Table 2. The X-ray density of the prepared samples was calculated using this formula [24]:

**Table 2:** X-ray density, apparent density, porosity, and FT-IR spectral data of Ni_0.7-*x*_Cu_0.3_Zn_*x*_Fe_2_O_4 _ferrite nanoparticles

Zn content	X-ray density	Apparent density	Porosity	Vibrational modes	
(*x*)	*D_hkl _*(nm)	*D_x _*(g cm^-3^)	*P *(%)	*v*_1_(cm^-1^)	*v*_2_(cm^-1^)
0.00	5.5030	5.2095	5.33	611	421
0.05	5.4969	5.2199	5.35	613	421
0.10	5.4780	5.1810	5.42	613	422
0.15	5.4730	5.1695	5.54	615	421
0.20	5.4653	5.1486	5.79	617	421

(7) $$D_{hkl}=\frac{ZM}{Na^{3}},$$

where *Z *is the number of molecules per unit cell (*Z *= 8), *M *is the molecular weight, *a *is the lattice parameter, and *N *is the Avogadro's number. The theoretical density of the samples was calculated using this formula [24]:

(8) $$D_{x}\text{ = }\frac{m}{Vr^{2}h},$$

where *m*, *V*, *r*, and *h *are the mass, volume, radius, and thickness of the samples, respectively. The porosity of the samples was calculated using this formula:

(9) $$P\text{ = 1}-\frac{D_{x}}{D_{hkl}}\times 100.$$

The apparent density of the specimens is about 94% to 95% of the corresponding X-ray densities. The data in Table 2 show that both densities decrease with increasing Zn content, i.e., the apparent density nearly reflects the same general behavior with the X-ray density.

The increase of porosity and decrease of shrinkage with increasing Zn^2+ ^ion content are related to the rapid densification of ferrite samples and also to the difference in specific gravity of the ferrite components since NiO (6.72 g cm^-3^) is heavier than ZnO (5.6 g cm^-3^) [25]. Also, it is known that the porosity of ceramic samples is a result that came from two sources: the intragranular porosity [*P*_intra_] and intergranular porosity [*P*_inter_] [26]. Thus, the total porosity *P *(in percent) could be written as the sum of the two types:

(10) $$P\left(\%\right)\;=\;\left(P_{\text{intra}}+\;P_{\text{inter}}\right).$$

Furthermore, it is reported that *P*_inter _depends on the grain size [27]. By the study of XRD and transmission electron microscopy [TEM] data of the samples, it is found that as the Zn concentration increases from *x *= 0.0 up to *x *= 0.2, there is no major change in the grain size. Therefore, as Zn^2+ ^ion content increases, *P*_inter _remains almost constant. Thus, according to Equation 10, the increase of the total porosity *P *(in percent) is mainly due to the increase of *P*_intra _with Zn^2+ ^doping [28,29].

**Figure 2:**
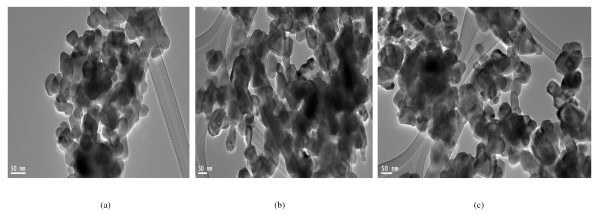
**TEM micrographs of (a) 0.0, (b) 0.5, and (c) 0.1 compositions of Ni_0.7-*x*_Zn_*x*_Cu_0.3_Fe_2_O_4 _ferrite nanoparticles**.

**Figure 3:**
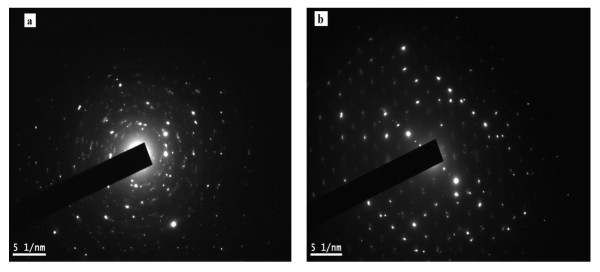
**SAED patterns of (a) 0.0 and (b) 0.5 compositions of Ni_0.7-*x*_Zn_*x*_Cu_0.3_Fe_2_O_4 _ferrite nanoparticles**.

**Figure 4:**
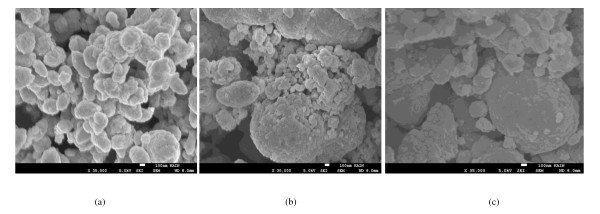
**SEM micrographs of (a) 0.0, (b) 0.5, and (c) 0.1 compositions of Ni_0.7-*x*_Zn_*x*_Cu_0.3_Fe_2_O_4 _ferrite nanoparticles**.

The FETEM and FESEM micrographs of the synthesized nanoparticles along with the selected area electron diffraction [SAED] pattern for pure and doped Ni-Cu-Zn ferrite nanoparticles are shown in Figures 2a, b, c, 3a, b, and 4a, b, c. The micrographs show largely agglomerated nanoparticles of the sample powder. An overview of the TEM image of nanoparticles shows that the particles have a size distribution of 28 to 32 (± 1) nm. The average size of the agglomerates is found to be 30 nm. Such aggregate formation and broader size distribution are characteristic of mechanically activated nanosized particles. The agglomeration of particles is also because they experience a permanent magnetic moment proportional to their volume [30]. Very few large particles having a size at approximately 40 nm have also been observed. It is clear from Table 1 that the particle size obtained from FETEM measurements corroborates well with the crystallite size obtained from XRD analysis. The shape of majority of the particles appears to be non-spherical. In the SAED image of synthesized nanoparticles, distinct rings that confirm good crystallinity are clearly visible. The observed crystallographic *d *values of 2.52 Å correspond to the lattice space of (311) plane of the Ni-Cu-Zn ferrite system. The observed crystallographic *d *values agree well with those obtained from XRD analysis. The results of the XRD and TEM study divulge that all the samples are well crystalline-nanosized spinel ferrites. The average particle diameter was found to be 29 nm which agrees well with that estimated from XRD data.

### FT-IR measurements

**Figure 5:**
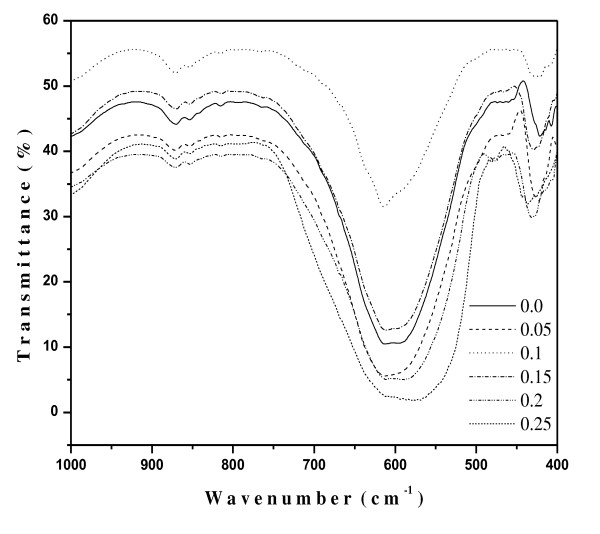
**FTIR spectra of Ni_0.7-*x*_Zn_*x*_Cu_0.3_Fe_2_O_4 _(0.0 ≤ *x *≤ 0.2, *x *= 0.05) ferrite nanoparticles**.

FT-IR spectra of the as-synthesized ferrite nanoparticles measured in the frequency range of 400 to 1,000 cm^-1 ^are shown in Figure 5. Two prominent absorption bands (*v*_1 _and *v*_2_) around 400 and 600 cm^-1^, respectively, are observed. These spectra represent characteristic features of ferrospinels, and bands are attributed to the stretching vibration due to interactions between the oxygen atom and the cations in tetrahedral and octahedral sites, respectively. The difference between *ν*_1 _and *ν*_2 _is due to the changes in bond length (Fe-O) at the octahedral and tetrahedral sites.

The FT-IR spectroscopic results are summarized in Table 3. From the table, it is clear that only the *v*_1 _band is perturbed with the incorporation of Zn ions in the Ni-Cu matrix. Significant changes were observed in the *v*_1 _band (corresponds to tetrahedral site), while no prominent perturbation was observed in the *v*_2 _band (corresponds to octahedral site). The frequency of the *v*_1 _band was observed to change from low frequency to higher frequency with progressive doping. The behavior is attributed to the stretching of Fe-O bonds on substitution of Zn ions. FT-IR results clearly indicate that Zn ions occupy the tetrahedral site in the Ni-Cu matrix of nanoparticles.

**Table 3:** Impedance parameters calculated from the complex impedance plots for various compositions at room temperature

Composition	*R*_gb _(calculated)	*R*_gb _(observed)	*C*_gb _(calculated)	*C*_gb _(observed)	Error
(*x*)	(KΩ)	(KΩ)	(F)	(F)	(%)
0.00	9.5562	9.485	3.9E - 2	4.3E - 3	0.05
0.05	8.8615	8.93	2.2E - 3	2.5E - 3	0.03
0.10	3.5984	3.643	9.5E - 3	9.9E - 3	0.03
0.15	78.794	77.27	6.7E - 3	6.5E - 3	0.05
0.20	80.032	81.23	5.2E - 4	5.1E - 4	0.01

### Electrical measurements

#### Dielectric measurements

The dielectric constant [*ε*'] of the samples was calculated using this formula:

(11) $$\varepsilon \prime =\frac{C_{\text{p}}t}{\varepsilon_{0}A},$$

where *ε*_0 _= 8.854 × 10^-12 ^F/m, known as permittivity of free space, and *t *is the thickness of the pellet. *A *is the area of cross section of the pellet, and *C*_p _is the capacitance of the pellet. The complex dielectric constant was calculated from this relation:

(12) ε"=ε′tanδ.

**Figure 6:**
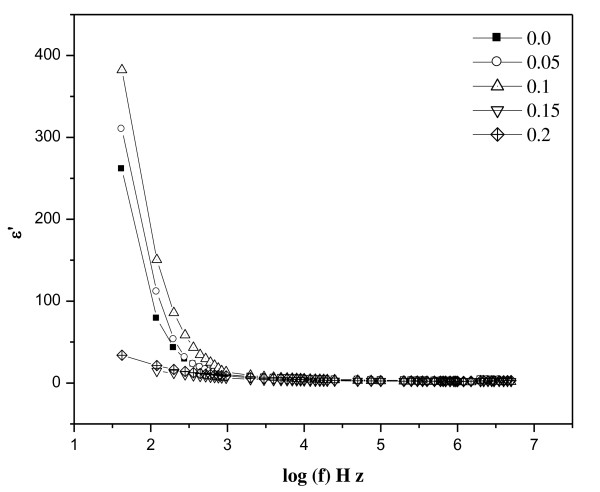
**The real part variation of the dielectric constant (*ε*') with frequency at room temperature**.

**Figure 7:**
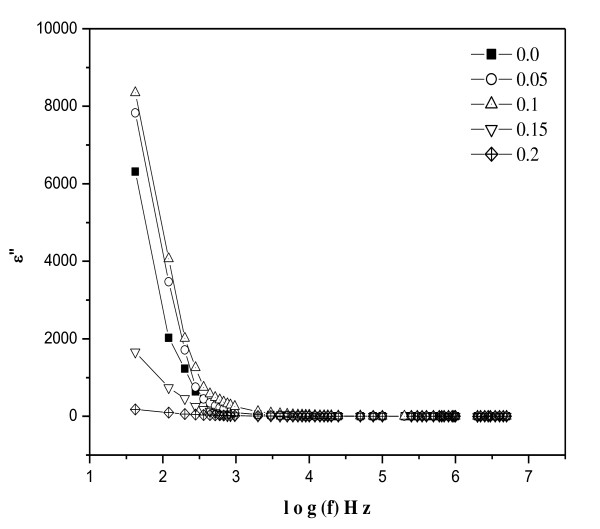
**The imaginary part variation of the dielectric constant (*ε*″) with frequency at room temperature**.

The frequency dependence of the dielectric constant for all the samples was studied at room temperature. Figures 6 and 7 depict the variation of real and complex parts of the dielectric constant with frequency. It is clear that all the studied samples exhibit dielectric dispersion where the values of both real (*ε*') and imaginary (*ε*″) parts of the dielectric constant decrease with increasing frequency of the field. The data reveal that none of the samples exhibit any anomalous behavior or peaking behavior. The observed dielectric behavior can be explained in the light of space charge polarization and hopping model [31-33].

The presence of Fe^3+ ^and Fe^2+ ^ions render ferrite materials to be dipolar. Polarization is also affected by factors such as structural homogeneity, stoichiometry, density, grain size, and porosity of the ferrites. The rotational displacement of dipoles results in orientational polarization. In ferrites, rotation of Fe^2+ ^to Fe^3+ ^and vice versa can be visualized as the exchange of electrons between two ions so that the dipoles align themselves with respect to the applied field. The polarization at lower frequencies may result from electron hopping between Fe^3+ ^⇔Fe^2+ ^ions in the ferrite lattice. The polarization decreases with increasing frequency and reaches a constant value due to the fact that beyond a certain frequency of external field, the electron exchange Fe^3+ ^⇔ Fe^2+ ^cannot follow the changes in the applied field. Also, the presence of Ni^3+^/Ni^2+ ^ions, which give rise to *p*-type carriers, contributes to the net polarization though it is small. The net polarization increases initially and then decreases with increasing frequency [34].

#### Dielectric loss

**Figure 8:**
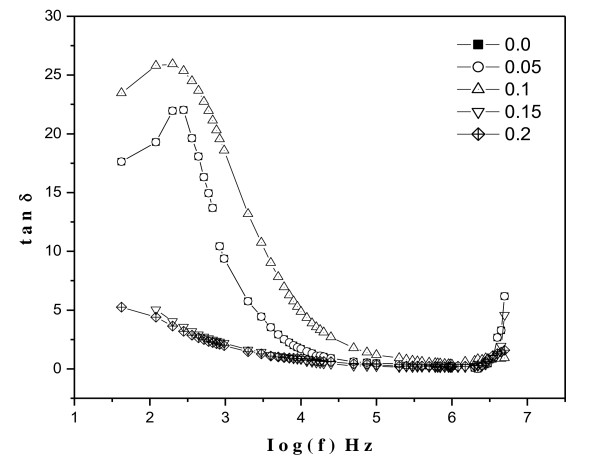
**The loss tangent (tan*δ*) variation with frequency at room temperature**.

Figure 8 shows the variation of dielectric loss tangent (tan*δ*) with frequency (42 Hz to 5 MHz) at room temperature. The dielectric loss decreases with the increasing frequency which is a normal behavior of any ferrite material. The dielectric loss decreases rapidly in the low-frequency region, while the rate of decrease is slow in the high-frequency region, and it shows an almost frequency independent behavior in the high-frequency region. The low loss values at higher frequencies show the potential applications of these materials in high-frequency microwave devices. The behavior can be explained on the basis that in the low-frequency region, which corresponds to a high resistivity (due to the grain boundary), more energy is required for electron exchange between Fe^2+ ^and Fe^3+ ^ions; as a result, the loss is high. In the high-frequency region, which corresponds to a low resistivity (due to the grains), small energy is required for electron transfer between the two Fe ions at the octahedral site. Moreover, the dielectric loss factor also depends on a number of factors, such as stoichiometry, Fe^2+ ^content, and structural homogeneity, which in turn depend upon the composition and sintering temperature of the samples [35,36].

#### ac Conductivity

**Figure 9:**
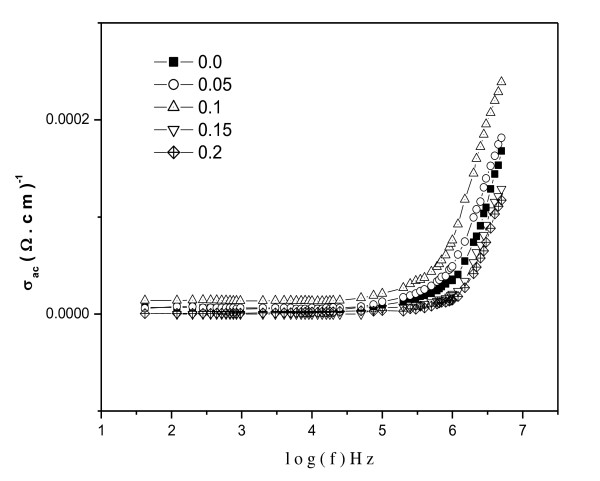
**The *σ*_ac _variation with frequency at room temperature**.

The ac part of the electrical conductivity was calculated from this relation:

(13) $$\sigma_{\text{ac}}=\varepsilon \prime \varepsilon_{0}\omega \text{tan}\delta ,$$

where *ω *is the angular frequency. Figure 9 shows the variation of ac conductivity with frequency (42 Hz to 5 MHz) at room temperature. The ac conductivity increases with increasing frequency above 200 KHz. Before that, it shows an almost frequency-independent behavior. The electrical conductivity in ferrites is mainly due to the hopping of electrons between ions of the same element present in more than one valence state and distributed randomly over crystallographic equivalent lattice sites. Ferrites have a cubic close-packed oxygen lattice with the cations at the octahedral (B) and tetrahedral (A) sites. The distance between two metal ions on the B sites is smaller (0.292 nm) than the distance between two metals ions on the A sites (0.357 nm). Therefore, the hopping between A⇔B sites has a very small probability compared with that at the B⇔B sites. The hopping between A⇔A sites does not exist due to the fact that there are only Fe^3+ ^ions at the A sites, and any Fe^2+ ^ions formed during the sintering process preferentially occupy the B sites only [37]. The charges migrate under the influence of the applied field, contributing to the electrical response of the system.

The conductivity is an increasing function of frequency in the case of conduction by hopping and a decreasing function of frequency in the case of band conduction [38]. The conductivity of a semiconductor material *σ *can be expressed as:

(14) $$\sigma \text{ = }\sigma_{\text{dc}}+\sigma_{\text{ac}}.$$

The first term (*σ*_dc_) is the direct current [dc] conductivity, which is due to band conduction, and it is frequency independent. The second term (*σ*_ac_) is the pure ac conductivity, which is due to the hopping processes at the octahedral site. The ac conductivity follows the empirical formula of the frequency dependence given by the ac power law [39]:

(15) $$\sigma \left(\omega \right)=B\omega^{n},$$

where *B *and *n *are constants which depend both on temperature and composition; *n *is dimensionless, whereas *B *has units of electrical conductivity.

In the present study, the conduction mechanism is due to electron hopping between Fe^2+^⇔Fe^3+ ^ions and hole hopping between Ni^2+^⇔Ni^3+ ^at two adjacent B sites. The charge exchange frequency increases with increasing frequency of the applied field, but the charge exchange mechanism does not follow the frequency of applied field beyond a certain frequency limit because at high frequencies, the resistivity remains invariant with the frequency, and as a result, the hopping frequency no longer follows the changes of external field beyond a certain frequency limit and thus lags behind [36].

**Figure 10:**
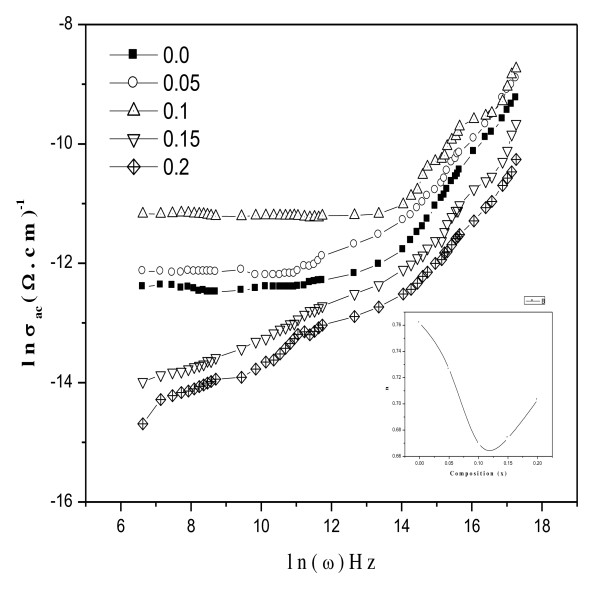
**The variation of ln*σ *versus ln*ω *with inset showing variation of exponent *n *with composition**.

Figure 10 shows the variation of ln *σ *versus ln *ω *in the frequency range of 42 Hz to 5 MHz at room temperature, with the inset showing the variation of exponent *n *with composition. The exponent *n *was calculated as a function of composition by plotting ln *σ *versus ln *ω *according to Equation 15, which represents straight lines with the slope equal to the exponent *n *and the intercept equal to ln *B *on the vertical axis at ln *ω *= 0. It is well known that *n *takes values between 0 and 1. When *n *= 0, the electrical conduction is frequency independent or becomes the dc conduction, but when *n *> 0, the conduction is frequency dependent or becomes the ac conduction [40]. In the present study, the value of *n *varies between 0.073 and 0.086, which suggests that the conduction phenomenon in the studied samples is ac conduction and is due to the hopping of charges.

#### Composition dependence of dielectric properties

**Figure 11:**
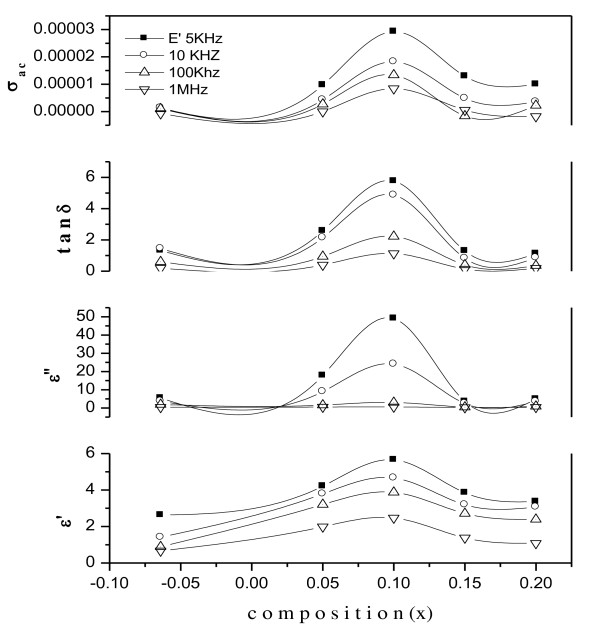
**The variation of *ε*', *ε*″, tan*δ*, and *σ*_ac _with Zn composition at room temperature**.

Figure 11 shows the variation of *ε*', *ε*″, tan*δ*, and *σ*_ac _with composition at selected frequencies. All the investigated electrical parameters show their maximum value for Ni_0.6_Cu_0.3_Fe_2_Zn_0.1_O_4 _composition. The composition-dependent behavior of the investigated samples can be explained on the basis that Ni as well as Cu ferrites are inverse spinel in structure, and the degree of inversion depends upon the heat treatment [41,42].

In the investigated samples, the presence of Ni^3+^/Ni^2+ ^ions leads to the formation of the *p*-type charge carriers, and their local displacement in the direction of applied field also contributes to net polarization, in addition to the *n*-type charge carriers. However, the contribution of the *p*-type carriers is small as compared with the electron exchange between Fe^3+^⇔Fe^2+ ^and is directed opposite to the flow of the *n*-type carriers [43]. It is believed that the hopping of electrons between Fe^2+^⇔Fe^3+ ^(*n*-type semiconductor) and the hopping of holes between Ni^3+^⇔Ni^2+ ^(*p*-type semiconductor) are responsible for the conduction process and dielectric polarization of the studied samples [44,45]. The maximum values of *ε*', *ε*″, tan*δ*, and *σ*_ac _for *x *= 0.1 can be explained on the basis that Zn^2+ ^ions doped in the Ni-Cu ferrite occupy the A sites, where the Fe^3+ ^ions present are forced to migrate from the A sites to the B sites. The increase in number of Fe^3+ ^ions at the B sites increases the rate of hopping which in turn increases the conductivity values for the composition *x *= 0.1, whereas the decrease in conductivity beyond *x *= 0.1 may be explained on the basis that further doping of Zn^2+ ^ions beyond *x *= 0.1 replace the Fe^3+ ^ions at the B sites which depletes the number of Fe ions available for the conduction phenomena, hence, the decrease in probability of following the exchange process:

(16) $$Ni^{2+}+Fe^{3+}↔Ni^{3+}+Fe^{2+}$$

Since dielectric polarization in ferrites is similar to electrical conduction, it is therefore expected that the behavior of *ε*', *ε*″, and tan*δ *is similar to that of *σ*_ac _for *x *= 0.1.

#### Impedance spectroscopy

It is well known that impedance spectroscopy is an important method to study the electrical properties of ferrites since impedance of the grains can be separated from the other impedance sources, such as impedance of electrodes and grain boundaries. One of the important factors, which influence the impedance properties of ferrites, is the nano- or microstructural effect. The complex impedance measurement gives us information about the resistive (real part) and reactive (imaginary part) components in the material. The complex impedance plot known as the Cole-Cole plot can give three semicircles, depending upon the electrical properties of the material. Since the ionic polarization in ferrites is not present, as a result, we have only two semicircles because of the space charge and orientation polarization in the ferrite materials. The first semicircle at low frequency represents the resistance of grain boundary. The second one obtained for high-frequency domain corresponds to the resistance of grain or bulk properties [46,47].

**Figure 12:**
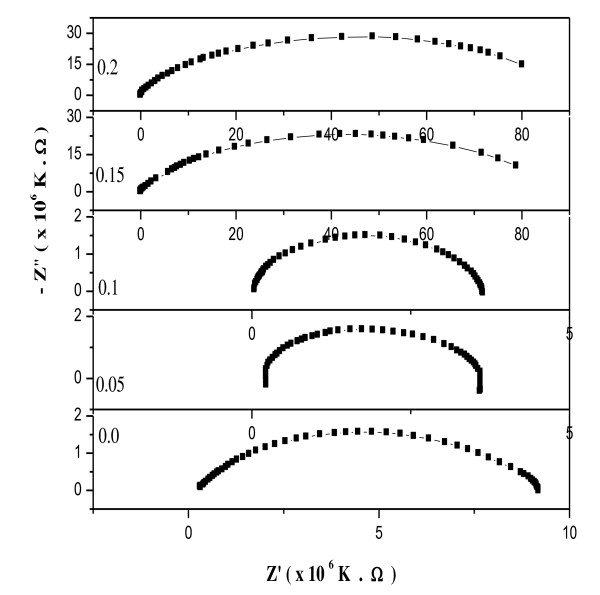
**The Cole-Cole plot of Ni_0.7-*x*_Cu_0.3_Zn_*x*_Fe_2_O4 ferrite nanoparticles at room temperature**.

Figure 12 shows the complex impedance plot for the different compositions of Ni-Cu-Zn ferrite nanoparticles taken at room temperature in the frequency range of 42 Hz to 5 MHz. The grain boundary (*R*_gb_), the grain resistance (*R*_g_), and the capacitance of grain and grain boundary (*C*_gb _and *C*_g_) were calculated at room temperature by analyzing the data using the nonlinear least square fitting routine and are presented in Table 3. The resistances were calculated from the circular arc intercepts on the *Z*'-axis, while the capacitance values were derived from the height of the circular arcs [48-50]. The plot obtained shows only one semicircular arc corresponding to the conduction due to the grain boundary volume in the low-frequency region, which suggests that conduction mechanism takes place predominantly through the grain boundary volume. Furthermore, the contribution from the grain was not well resolved in the samples. The higher value of the grain boundary can be due to the decrease in Fe^3+ ^number, increase in surface-to-volume ratio, porosity, and disordered atomic arrangement near the grain boundary.

## Conclusion

Nanoparticles of polycrystalline Ni_0.7-*x*_Cu_0.3_Fe_2_Zn_*x*_O_4 _ferrites, with an average crystallite size between 28 and 32 nm, were synthesized through auto-combustion method. The dielectric constant and loss tangent both show a normal behavior with respect to frequency. The dielectric and ac conductivity parameters show their maximum value for 10% Zn-doping composition. The overall resistance has been found solely in grain boundary volume, and the contribution of the grain is not well resolved. As a result, the conduction process predominantly takes place through the grain boundary.

## Competing interests

The authors declare that they have no competing interests.

## Authors' contributions

KMB and MSA were involved with the whole research work presented here. Both the authors have synthesized the nanoparticles and performed different experiments. All authors read and approved the final manuscript.
